# Head-to-head comparison between the EQ-5D-5L and the EQ-5D-3L in general population health surveys

**DOI:** 10.1186/s12963-018-0170-8

**Published:** 2018-08-16

**Authors:** Marc Martí-Pastor, Angels Pont, Mónica Ávila, Olatz Garin, Gemma Vilagut, Carlos G. Forero, Yolanda Pardo, Ricard Tresserras, Antonia Medina-Bustos, Oriol Garcia-Codina, Juan Cabasés, Luis Rajmil, Jordi Alonso, Montse Ferrer

**Affiliations:** 1 IMIM (Hospital del Mar Medical Research Institute), Health Services Research Group, Doctor Aiguader, 88, 08003 Barcelona, Spain; 2 CIBER en Epidemiología y Salud Pública (CIBERESP), Madrid, Spain; 3 Universitat Autonoma de Barcelona (UAB), Barcelona, Spain; 4 Universidad Pompeu Fabra, Barcelona, Spain; 5 Direcció General de Planificació en Salut. Departament de Salut de la Generalitat de Catalunya, Barcelona, Spain; 6 Public University of Navarra, Pamplona, Spain; 7 Agency for Healthcare Quality and Assessment of Catalonia (AQuAS), Barcelona, Spain

**Keywords:** Quality of life, Health survey, Validity and reliability, Perceived health

## Abstract

**Background:**

The EQ-5D has been frequently used in national health surveys. This study is a head-to-head comparison to assess how expanding the number of levels from three (EQ-5D-3L) to five in the new EQ-5D-5L version has improved its distribution, discriminatory power, and validity in the general population.

**Methods:**

A representative sample (*N* = 7554) from the Catalan Health Interview Survey 2011–2012, aged ≥18, answered both EQ-5D versions, and we evaluated the response redistribution and inconsistencies between them. To assess validity of this redistribution, we calculated the mean of the Visual Analogue Scale (VAS), which measures perceived health. The discriminatory power was examined with Shannon Indices, calculated for each dimension separately. Spanish preference value sets were applied to obtain utility indices, examining their distribution with statistics of central tendency and dispersion. We estimated the proportion of individuals reporting the best health state in EQ-5D-5L and EQ-5D-3L within groups of specific chronic conditions and their VAS mean.

**Results:**

A very small reduction in the percentage of individuals with the best health state was observed, from 61.8% in EQ-5D-3L to 60.8% in EQ-5D-5L. In contrast, a large proportion of individuals reporting extreme problems in the 3 L version moved to severe problems (level 4) in the 5 L version, particularly for pain/discomfort (75.5%) and anxiety/depression (66.4%). The average proportion of inconsistencies was 0.9%. The pattern of the perceived health VAS mean confirmed the hypothesis established a priori, supporting the validity of the observed redistribution. Shannon index showed that absolute informativity was higher in the 5 L version for all dimensions. The means (SD) of the Spanish EQ-5D-3L and EQ-5D-5L indices were 0.87 (0.25) and 0.89 (0.22). The proportion of individuals with the best health state within each specific chronic condition was very similar, regardless of the EQ-5D version (≤ 30% in half of the 28 chronic conditions).

**Conclusion:**

Although the proportion of individuals with the best possible health state is still very high, our findings support that the increase of levels provided by the EQ-5D-5L contributed to the validity and discriminatory power of this new version to measure health in general population, as in the national health surveys.

**Electronic supplementary material:**

The online version of this article (10.1186/s12963-018-0170-8) contains supplementary material, which is available to authorized users.

## Background

Health-related quality of life has been gaining importance in research, clinical practice and health planning [1, 2] by providing complementary information to health indicators based on morbidity and mortality. This is especially relevant to describe health in developed countries, where life expectancy has been increasing steadily after their epidemiological transition. Evaluating the general population’s health is one of the specific applications proposed for health-related quality of life instruments [3].

The EQ-5D has been frequently selected for national health surveys [4–10], given its low respondent burden and its consistently proven metric properties [6, 11, 12] . However, the high percentage of individuals with the best health state in the EQ-5D [13, 14] has been repeatedly highlighted as a limitation, since this may reduce its capacity to discriminate within good health [6, 15, 16], and its responsiveness in some health areas [17–19]. The traditional EQ-5D descriptive system, composed of five dimensions (mobility, self-care, usual activities, pain/discomfort, and anxiety/depression) with three levels of severity, defines 243 distinct health states [20] resulting from all the possible combinations (i.e., 3^5^). This is a very low number compared with other instruments, such as the Health Utilities Index [21] with 972,000 or the SF-6D [22] with 18,000 possible combinations.

To improve its discriminative capacity and sensitivity to change, and to reduce ceiling effects, the EuroQol Research Foundation decided to develop a new EQ-5D version increasing the number of response options from three (EQ-5D-3L) to five levels (EQ-5D-5L), resulting in 3125 health states (i.e., 5^5^). Face and content validity of the new EQ-5D-5L were demonstrated for both the English and Spanish versions through focus group research [23]. Studies performed in cancer [24, 25], hepatitis B [26], or hip arthroplasty [27] patients showed improvements for discriminative capacity [24, 26], construct validity [24–26], and responsiveness, without diminishing its reliability [25], as well as a large decrease in the percentage of individuals with the best health state.

Given the recent development of the EQ-5D-5L, there are still few head-to-head studies in general population comparing its metric properties with the traditional 3 L version. Studies carried out in South Korea [28], Alberta (Canada) [29], England [30] and Lombardy (Italy) [31], mainly based on national health surveys, examined both versions of EQ-5D in general population. Yet the South Korean study published in 2013 [28] was performed only in a small sample (*n* = 600), neither the Canadian [29] nor the English health surveys [30] administered both versions together, while the Italian survey did, but without comparing them. The decrease in the percentage of individuals with the best health state varied in these studies, from 42.1 to 32.3% in Alberta [29], from 56.2 to 47.6% in England [30], from 43.9 to 38% in Lombardy [31], and from 65.7 to 61.2% in South Korea [28]. The aim of this study is a head-to-head comparison to assess to what extent expanding the number of levels in the EQ-5D from three to five has improved its distribution, discriminatory power, and validity in the general population.

## Methods

### Study population

Data used in this study came from the Catalan Health Interview Survey (CHIS), a continuous cross-sectional study carried out since 2010 in Catalonia [32], an Autonomous Community in the northeast of Spain with about seven million inhabitants. A representative sample of Catalonia’s non-institutionalized population, without any age limit, is surveyed through computer-assisted personal interviews administered by an accredited team of interviewers in the respondent’s home. The CHIS was approved by the Consultants’ Committee of Confidential Information Management at the Catalan Health Department, according to the 2000 revision of the Helsinki Declaration.

Information collection is based on an uninterrupted random sampling strategy divided into waves with 6 months of duration. Each wave has an independent subsample of around 2500 individuals of all ages (representative of the Autonomous Community population), and a complete cycle is composed of eight waves with around 20,000 participants interviewed over 4 years (representative of the healthcare-governing districts).

### Study design

The CHIS complex sampling process was designed to ensure the territorial representativeness of the sample in every wave, taking into account the distribution of the Catalan population. In a first stage, health care-governing districts were systematically selected. At a second stage, municipalities were chosen at random after stratifying by number of inhabitants. In a third stage, participants from each municipality were selected by simple random sampling from the Catalan census register, after stratifying by age and gender.

The two EQ-5D versions (3 L and 5 L) were included in four waves (2nd to 5th) of the CHIS, conducted from January 2011 to December 2012 (*N* = 9658). Both versions of EQ-5D were face-to-face, computer-assisted interviews, always administered in the same order: first the EQ-5D-3L and next the EQ-5D-5L, followed by the visual analogue scale. Furthermore, to assess the effect of administering the two versions of EQ-5D together, we used data from the 6th wave (the first one where EQ-5D-5L was administered alone) to compare with the 5th wave (the last one where the two EQ-5D versions were administered together).

To correct the effect of non-response, 49% of selected sampling units needed to be replaced by others with the same characteristics in terms of age group, sex, and neighborhood. Reasons for replacement were: refusal to participate (25.9%), change of address (34.7%), prolonged absence (17.8%), inaccessible dwelling (12.6%), wrong address (4.0%), language skills (0.6%), death (1.4%), or other reasons (3.0%).

### Study variables

The EQ-5D is a generic, multi-attribute health status measure composed of a descriptive system, and a visual analogue scale (VAS) asking individuals to rate their own health from 0 to 100 (the worst and best imaginable health, respectively). The descriptive system covers five dimensions of health, and response options include three or five levels of severity according to the version. In general, the grading terms for level 1 (no problems), and 5 (extreme problems/unable to) on the EQ-5D-5L are consistent with the extreme levels of the EQ-5D-3L, except for “confined to bed” (EQ-5D-3L) vs. “unable to walk about” (EQ-5D-5L). Label description on EQ-5D-5L is “slight” for level 2 and “severe” for level 4 (except for anxiety/depression, with “slightly” and “severely”). The Spanish value set of preferences elicited with Time Trade Off (TTO) was applied to construct the EQ-5D-3L index [33], while the EQ-5D-5L index was calculated with the crosswalk 3 L–5 L value set [34], derived from the original EQ-5D-3L preference weights [33]. This crosswalk 3 L–5 L value set was obtained using a non-parametric indirect model [34] to generate values for the 5 L by estimating the probabilities of being in each of the 3 L levels. Thus, the theoretical ranges of the EQ-5D-5L index calculated with the crosswalk value set matched exactly with the 3 L index: from 1 (the best health state) to − 0.65 (negative values in health states valued as worse than death), where 0 is equal to death.

Sociodemographic variables recorded in the survey included gender, age, level of education, and social class. Social class was assigned according to the respondent’s most recent occupation (or the head of the household’s occupation in the case of those who were looking after the home), using an adapted version of the British Registrar General Social Classes: classes I and II (managerial and freelance professionals), class III (skilled non-manual occupations), class IV (skilled manual workers), and class V (non-skilled manual workers) [35].

Health indicators collected in the CHIS included general perceived health (rated as excellent, very good, good, fair or poor), limitation of daily activities due to any health problem during the previous 6 months, and a checklist of 28 common chronic conditions. Respondents were asked, “Do you suffer from or have you suffered from any of the following chronic conditions?” and had to answer “Yes” or “No” for each condition. A summary indicator was derived from the checklist, based on the number of reported chronic conditions. This discrete variable was categorized according to sample distribution into five groups: none, 1, 2, 3–4, and 5 or more chronic conditions.

### Statistical analysis

The sample size of CHIS allows calculating the proportion of individuals with the best health state among those reporting stroke (the least prevalent condition among the Catalan population) for an estimated percentage of 20% with a 95% confidence interval of +/− 5.

To restore the representativeness of the Catalan population, taking into account the complex sampling process followed (considering age, gender, and municipality), a weighting factor was applied. In addition, design-based standard errors and significance tests were estimated with the Taylor series linearization method implemented in SAS software, which account for the correlation structure among individuals induced by the stratified and clustered sampling design [36]. In order to determine the effect of the sampling in the estimations, the design effects were obtained as the ratio between two variances: the variance of the estimator under the actual sample design to that under simple random sampling of the same size.

Sample characteristics were described by calculating unweighted frequencies and weighted percentages. To evaluate the response redistribution between the classical EQ-5D and the new five level version, we first calculated weighted percentages in each level of the EQ-5D-5L after stratifying by responses to the EQ-5D-3L and, second, we assessed the inconsistencies according to the method described by Janssen et al. [37]. Briefly, from the 15 potential 3 L–5 L response pairs in each dimension, those skipping the adjacent categories of the 5 L were defined as inconsistencies. To assess validity of the response redistribution between three and five levels, we calculated the mean of the perceived health VAS in each of these 15 subgroups of potential pairs. Our hypothesis is that, except for inconsistencies, the perceived health (VAS) in subgroups of individuals selecting an EQ-5D-5L category with more severe problems is worse than in subgroups remaining in the same category of response to the EQ-5D-3L (or vice versa, better perceived health in milder problems).

The discriminatory power was examined with Shannon Indices, which were calculated for each dimension separately. The Shannon index is defined as:

$$ {H}^{\prime }=-\sum \limits_{i=1}^L{p}_i{\log}_2{p}_i $$

where H′ represents the absolute amount of informativity captured, L is the number of levels, and p_i_ = n_i_/N, the proportion of observations in the ith level (*i* = 1,…, L), n_i_ being the observed number of responses in level i and N the total sample size [38]. H′ reaches its maximum (H′ max) when distribution is uniform (rectangular) and it equals to log_2_ L. Shannon’s Evenness index (J’ = H′/H’max) reflects the evenness (spread) of a distribution, regardless of the number of levels. The 95% confidence intervals were calculated according to the variance of the Shannon index:

$$ \mathit{\operatorname{var}}\ {H}^{\prime }=\frac{\sum \limits_{i=1}^L{p}_i{\left({\mathit{\log}}_2{p}_i\right)}^2-{\left(\sum \limits_{i=1}^L{p}_i{\mathit{\log}}_2{p}_i\right)}^2}{\mathrm{N}} $$

As previously reported [37, 39, 40], we hypothesized that the 5 L has more discriminatory power (larger H′ values) than the 3 L version, but lower Shannon Evenness index J’, reflecting that populations need a larger spread to cover five levels than for three. Therefore, we expected the H′ to increase (higher absolute levels of information) and J’ to stay equal or marginally decrease in the 5 L version.

A plot between EQ-5D-3L index (*y*-axis) and EQ-5D-5L index (*x*-axis) was constructed to graphically compare the distribution of both indices. We also calculated the statistics describing the distribution of EQ-5D indices: the theoretical and observed ranges, the weighted proportion and 95% confidence intervals (95% CI) of individuals with the best and worst health state, and parameters of central tendency and dispersion. Furthermore, a sensitivity analysis was performed to examine the consistency of results when the EQ-5D-5L index is estimated with 3 L–5 L crosswalk value set or with the newly developed Spanish value set obtained through a common composite method of TTO and discrete choice experiments (DCE) [41]. We calculated the statistics describing the distribution of the EQ-5D-5L index constructed with this value set in the entire sample; as well as after excluding participants with negative values in any index, because the theoretical range of this new EQ-5D-5L index (− 0.416 to 1) was not exactly coincident with the EQ-5D-3L index (− 0.653 to 1) for values < 0.

To explore the distribution of EQ-5D indices in persons with chronic conditions, the weighted proportion (95% CI) of individuals reporting the best possible health state (11111) in EQ-5D-3L and EQ-5D-5L within each of the 28 specific chronic conditions’ groups was estimated. Furthermore, the mean (95% CI) of the perceived health VAS for this subgroup of individuals reporting the best possible health state within each specific chronic condition was calculated. Since we expected a lower proportion of individuals reporting the best health state (11111) with EQ-5D-5L than with EQ-5D-3L, we hypothesized a better perceived health (VAS) when this subgroup of individuals is defined by the EQ-5D-5L.

Finally, to assess the effect of administering the EQ-5D-5L after the 3 L version, we compared the responses to the dimensions in the EQ-5D-5L between the samples of the 5th (3 L and 5 L versions administered together) and 6th waves (EQ-5D-5L administered alone) using a Chi-squared test.

## Results

Of the 9658 participants in the CHIS between January 2011 and December 2012, 7554 individuals aged 18 to 102 years old were analyzed after excluding 2104 people younger than 18. Mean age of participants was 47.1 (SD = 18.9), and 50.9% were female (Table 1). More than half had completed secondary studies, 40% belonged to social class IV, and 48.5% suffered three or more chronic conditions. Only 15% of the individuals reported some limitation of activities in the previous 6 months, and 34.3% claimed to have either excellent or very good perceived health (Table 1).

**Table 1:** Sample characteristics of the Catalan Health Interview Survey (2011–2012)

	*n* (%) Unweighted	*n* (%) Weighted	SE^a^	Design effect
Gender				
Male	3791 (50.2%)	3877 (49.1%)	0.20	0.19
Female	3763 (49.8%)	4014 (50.9%)	0.20	0.19
Age group				
18–44 years	3527 (46.7%)	3801 (48.2%)	0.45	0.62
45–64 years	2259 (29.9%)	2436 (30.9%)	0.76	2.08
65–74 years	753 (10.0%)	784 (9.9%)	0.33	0.92
75 years and over	1015 (13.4%)	870 (11.0%)	0.29	0.53
Studies level				
Primary or less	2015 (26.7%)	1993 (25.3%)	2.19	18.52
Secondary	4179 (55.4%)	4345 (55.1%)	1.65	8.31
University or more	1356 (18.0%)	1548 (19.6%)	3.44	60.70
Social class				
I-II (managerial and free-lance professionals)	1312 (18.0%)	1485 (19.5%)	2.83	40.90
III (skilled non-manual occupations)	2226 (30.6%)	2390 (31.3%)	2.36	19.84
IV (skilled manual workers)	3067 (42.2%)	3052 (40.0%)	4.71	68.95
V (non-skilled manual workers)	671 (9.2%)	701 (9.2%)	0.59	3.18
Perceived health				
Excellent	564 (7.5%)	636 (8.1%)	0.82	7.41
Very good	1895 (25.1%)	2067 (26.2%)	1.64	10.84
Good	3388 (44.9%)	3452 (43.7%)	2.08	13.25
Fair	1356 (18.0%)	1374 (17.4%)	0.48	1.20
Poor	351 (4.7%)	362 (4.6%)	0.41	2.82
Activity limitation				
Yes, seriously affected	398 (5.3%)	397 (5.0%)	0.33	1.60
Yes, limited but not seriously	762 (10.1%)	786 (10.0%)	0.63	3.33
No	6394 (84.6%)	6708 (85.0%)	0.85	4.19
Number of chronic physical conditions				
None	1690 (22.4%)	1783 (22.6%)	1.60	11.21
1 condition	1183 (15.7%)	1256 (15.9%)	0.55	1.75
2 conditions	981 (13.0%)	1017 (12.9%)	0.50	1.66
3 or 4 conditions	1432 (19.0%)	1526 (19.3%)	0.47	1.07
5 or more conditions	2268 (30.0%)	2308 (29.2%)	1.36	6.68
VAS (mean, SD)	7554	73.19 (19.21)	0.42	5.21

^a^Standard error was estimated by the Taylor series method

Cross tabulations of responses to both EQ-5D versions (Table 2) showed that most of the participants reporting no problems in the 3L version remained at Level 1 in the 5L version, and only 1–2% moved to slight problems. In contrast, a large proportion of individuals reporting extreme problems in the 3L version had moved to severe problems (Level 4) in the 5L version. This proportion was particularly marked for pain/discomfort (75.5%) and anxiety/depression (66.4%). Grey cells show the pairs previously defined as inconsistencies. The number of inconsistencies was highest in the pain/discomfort domain (*n* = 189; 2.4%) and lowest in the self-care one (*n* = 54; 0.6%). The average proportion of inconsistencies by dimension was 0.9%.

**Table 2:** Comparison between EQ-5D-5L and EQ-5D-3L responses, and mean of perceived health VAS

	EQ-5D-5L				
EQ-5D-3L	No problems 1	Slight problems 2	Moderate problems 3	Severe problems 4	Unable/extreme 5
Mobility					
No problems in walking about (*n* = 6390)	6287 (98.6%) [77.4]	86 (1.2%) [58.5]	**16 (0.2%) [53.5]**	**1 (0.0%) [15.0]**	**0 (0.0%) -**
Some problems in walking about (*n* = 1104)	**36 (3.2%) [60.7]**	392 (34.8%) [57.0]	436 (41.1%) [48.9]	221 (19.8%)[38.2]	**19 (1.1%)[52.2]**
Confined to bed (*n* = 60)	**3 (4.4%) [74.5]**	**1 (0.2%) [40.0]**	**3 (7.9%) [37.3]**	15 (26.5%) [35.2]	38 (60.9%) [35.5]
Self-care					
No problems with self-care (*n* = 7057)	6956 (98.6%) [75.4]	88 (1.2%) [46.8]	**12 (0.2%) [32.5]**	**1 (0.0%) [40.0]**	**(0.0%) -**
Some problems washing or dressing myself (*n* = 345)	**27 (6.3%) [61.8]**	109 (29.1%) [49.9]	154 (48.9%) [43.7]	51 (14.9%) [30.6]	**4 (0.8%) [24.9]**
Unable to wash or dress myself (*n* = 152)	**2 (1.5%) [76.9]**	**3 (1.7%) [52.3]**	**5 (3.6%) [55.4]**	29 (18.4%) [44.7]	113 (74.9%) [36.5]
Usual activities					
No problems with performing my usual activities (*n* = 6677)	6526 (97.8%) [77.0]	105 (1.6%) [58.2]	**37 (0.5%) [56.5]**	**8 (0.1%) [36.3]**	**1 (0.0%) [70.0]**
Some problems with performing my usual activities (*n* = 600)	**31 (4.3%) [59.0]**	197 (31.3%) [53.8]	269 (46.3%) [46.0]	92 (16.3%) [40.0]	**11 (1.7%) [47.1]**
Unable to perform my usual activities (*n* = 277)	**1 (0.6%) [70.0]**	**2 (0.5%) [69.1]**	**20 (7.7%) [48.8]**	81 (30.0%) [42.2]	173 (61.3%) [35.0]
Pain/discomfort					
No pain or discomfort (*n* = 5275)	5124 (97.3%) [79.7]	113 (2.0%) [68.1]	**34 (0.6%) [65.5]**	**4 (0.1%) [65.9]**	**0 (0%) -**
Moderate pain of discomfort (*n* = 1846)	**73 (3.7%) [68.7]**	790 (41.9%) [67.6]	875 (47.9%) [59.4]	107 (6.6%) [49.4]	**1 (0.0%) [40.0]**
Extreme pain or discomfort (*n* = 433)	**0 (0%) -**	**7 (1.8%) [55.8]**	**70 (15.7%) [47.9]**	324 (75.5%) [40.1]	32 (7.0%) [34.2]
Anxiety/depression					
Not anxious or depressed (*n* = 6226)	6098 (98.1%) [77.4]	100 (1.5%) [61.0]	**21 (0.3%) [65.8]**	**6 (0.0%) [43.9]**	**1 (0.0%) [50.0]**
Moderately anxious or depressed (*n* = 1111)	**52 (4.5%) [58.0]**	526 (47.0%) [62.1]	474 (43.6%) [54.8]	56 (4.6%) [46.1]	**3 (0.3%) [22.3]**
Extremely anxious or depressed (*n* = 217)	**3 (1.5%) [49.6**]	**6 (2.4%) [51.5]**	**37 (18.2%) [46.5]**	147 (66.4%) [41.7]	24 (11.5%) [29.5]

N unweighted, (weighted % by response to EQ-5D-3L) and [mean VAS]. Inconsistencies are marked in bold

Regarding the validity of the redistribution between three and five levels, the mean of the perceived health VAS was over 75 in the subgroup of individuals reporting no problems in both versions for all dimensions (range 75.4–79.7). Confirming the hypothesis established a priori, the perceived health VAS mean in subgroups of individuals selecting an EQ-5D-5L category of more severe problems is worse than in those remaining in the same category as in the EQ-5D-3L. Similarly, those moving to milder problems in the EQ-5D-5L presented better perceived health. For example, in the last row of Table 2 (extremely anxious or depressed in the EQ-5D-3L), the 66.4% who moved to a milder level in the 5 L (severe problems) presented better perceived health than those who remained at the extreme level (11.5%): mean VAS of 41.7 vs. 29.5.

**Fig. 1:**
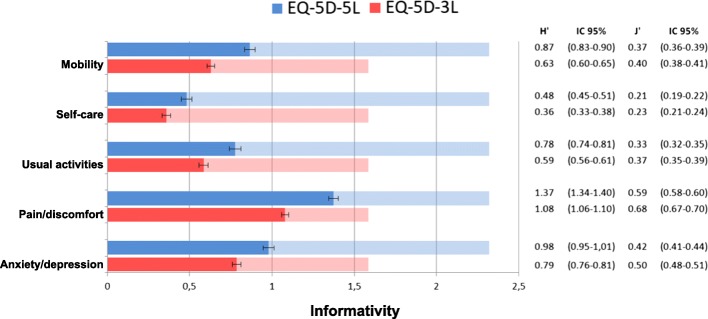
Discriminatory power measured by Shannon Indices for 3 L and 5 L version. Footnote: Absolute Informativity (H′) represented by dark bars and Maximum Absolute Informativity (H’max) represented by light bars. The Relative Informativity (J’) is the proportion of H′/H’max

Figure 1 shows Shannon indices of EQ-5D-3L and EQ-5D-5L. The maximum information captured by the system (H’max in light bars), and also the absolute informativity (H′ in dark bars) is higher in 5 L than in 3 L version. However, when H′ is compared with the H’max, the relative information area captured (J’) is significantly lower in EQ-5D-5L than in 3 L for all dimensions except self-care. This difference is especially marked in pain/discomfort (J’ = 0.59 vs. 0.68) and anxiety/depression (J’ = 0.42 vs. 0.50).

**Fig. 2:**
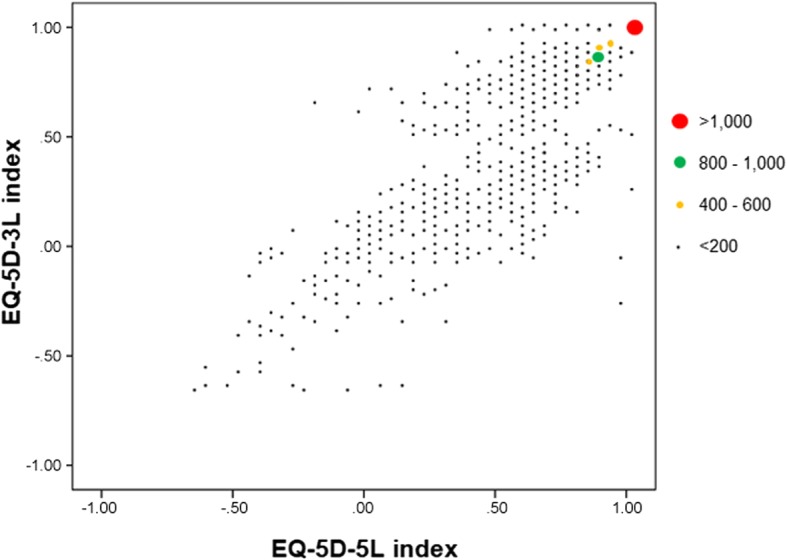
Plot between EQ-5D-3L and EQ-5D-5L indices. Footnote: The EQ-5D-3L index was calculated with the conventional Time Trade Off preference values from the Spanish general population [33]; and the EQ-5D-5L index was calculated with the 3 L–5 L crosswalk from Spain [34]

Figure 2 shows the plot between EQ-5D-3L and EQ-5D-5L indices. The cloud of points and the biggest clusters of individuals were concentrated around the perfect agreement diagonal, indicating a high correlation between both indices. A slight deviation to higher values with the EQ-5D-5L than the EQ-5D-3L is also observable.

Table 3 shows the statistics describing distribution of the EQ-5D indices. Ranges observed in our sample matched exactly with the theoretical ranges (from −0.65 to 1). The proportion of individuals with the worst health state was negligible (< 0.15%), while the proportion with the best health state was 61.8% with EQ-5D-3L and 60.8% with EQ-5D-5L. Means (SD) were 0.87 (0.25) and 0.89 (0.22) for EQ-5D-3L and EQ-5D-5L. Sensitivity analysis performed with the EQ-5D-5L index constructed with the newly developed Spanish value set [41] (see Additional file 1) showed consistent results: mean 0.90 (SD = 0.19) in the entire sample, and mean 0.92 (SD = 0.14) after excluding the 249 subjects with negative values. Differences between EQ-5D-3L and EQ-5D-5L indices remained quite stable regardless the value set used.

**Table 3:** Distribution of the EQ-5D-3L and EQ-5D-5L indices (total sample and positive values subsample^a^)

	EQ-5D-3L	EQ-5D-5L
Total sample	*N* = 7554	*N* = 7554
Theoretical range	−0.653, 1	− 0.654, 1
Observed range	− 0.653, 1	−0.654, 1
% with worst health state (95% CI)	0.14% (0.04, 0.24)	0.03% (0, 0.08)
% with best health (95% CI)	61.82% (59.38. 64.26)	60.82% (58.36, 63.28)
Mean, SD (95% CI)	0.87, SD = 0.25 (0.86, 0.88)	0.89, SD = 0.22 (0.88, 0.90)
Median [IQR]	0.93 [0.87, 0.96]	0.94 [0.88, 0.97]

^a^After excluding participants with negative values in any index

The EQ-5D-3L index was calculated with the conventional Time Trade Off preference values from the Spanish general population [33]; and the EQ-5D-5L index was calculated with the 3 L–5 L crosswalk from Spain [34]

**Fig. 3:**
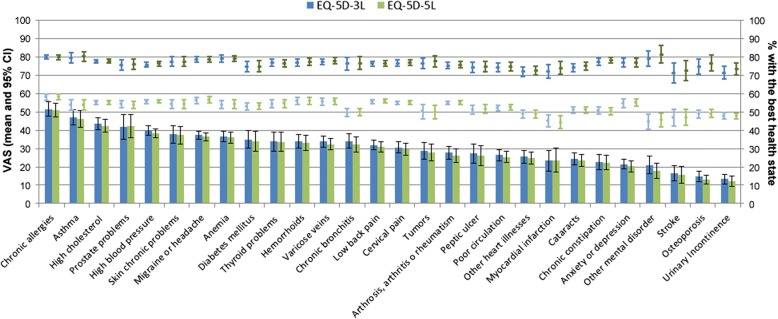
EQ-5D-3L (blue) and EQ-5D-5 L (green): Individuals with best health state within each chronic condition. Footnote: Bars show weighted proportions and 95% CI of individuals with best health (11111). Lines show mean of VAS and 95% CI: best possible health, 11111 (dark); some health problem (light)

Figure 3 shows results by each specific chronic condition: the proportion of individuals with the best health state (11111) in the EQ-5D-3L (blue bars) and EQ-5D-5L (green bars), and also the mean (95% CI) of perceived health VAS in subgroups with and without the best health state. In both indices, chronic allergies presented the highest proportion of subjects with the best health state (50.6 and 50.1%), and urinary incontinence the lowest (13.1 and 12.0%). Regardless of the index used, the proportion of individuals with the best health state was ≤ 30% in half of the chronic conditions from the checklist (cervical pain, tumors, arthrosis, arthritis or rheumatism, peptic ulcer, poor circulation, other health illnesses, cataracts, myocardial infarction, chronic constipation, anxiety or depression, other mental disorders, stroke, osteoporosis, and urinary incontinence). The mean of the VAS for the subgroup with the best possible health state defined by EQ-5D-3L and EQ-5D-5L (in dark blue and green lines, respectively) was over 70 within all specific chronic condition groups, ranging 71.3–79.8 and 72.6–81.3, respectively. Perceived health VAS means in the subgroups defined by the EQ-5D-3L were very similar to those obtained in the subgroups defined by EQ-5D-5L. For the subgroup with some health problem (not 11111), mean of VAS was always lower than 60 (light blue and green lines).

## Discussion

This head-to-head comparison of EQ-5D-5L with EQ-5D-3L in the general population of Catalonia shows that redistribution of levels is mostly in individuals reporting extreme problems on the EQ-5D-3L, which moved to level 4 on the EQ-5D-5L, but not for those reporting no problem, who remained at the top. This explains the very small reduction in the percentage of individuals with the best health state, from 61.8% with EQ-5D-3L to 60.8% with EQ-5D-5L, and the increment of the index mean (from 0.87 to 0.89) in our sample.

One of the original contributions of this study is that, as far as we are aware, this is the first time that distribution and validity of the EQ-5D-5L have been compared head-to-head to those of the EQ-5D-3L in a health survey on general population. In the Lombardy study both versions were also administered, but they were not compared since the publication was focused on reference norms [31].

Our study has some limitations. Firstly, the two versions of the EQ-5D were always administered in the same order: first the 3 L and then the 5 L. This proximity might have affected the EQ-5D-5L, which was always administered second. However, the comparison with the 6th wave (see Additional file 2), where only the EQ-5D-5L was administered, showed no differences in EQ-5D-5L dimensions, except for pain/discomfort (72.4% versus 67.6% of individuals reporting no problem, *p* = 0.003). This finding indicates that the fact of administering the two versions together did not modify the response to the EQ-5D-5L when administered alone (as in the 6th wave). Furthermore, results from the 2011 National Health Survey of Spain (62.4% of individuals with the best health state) where only the 5 L version was administered [42] also support our EQ-5D-5 L findings in Catalonia. Secondly, an interviewer bias may have played a role, and this could be differential for those dimensions where the wording of the response option had been modified in the EQ-5D-5L. For example, in the extreme of mobility (“confined to bed” for the EQ-5D-3L versus “unable to walk about” for the EQ-5D-5L), interviewers might have attenuated the severity. Finally, our sample is only representative of Catalonia. However, given the similarities in national indicators such as life expectancy or healthy life years in the general population of Catalonia, Spain, and other European regions [43], it is likely that our results will be generalizable to similar developed countries.

The small reduction observed in the percentage of individuals with the best health state, from 61.8% with EQ-5D-3L to 60.8% with EQ-5D-5L, is due to the negligible movement from level 1 out of 3 (“no problem”) to level 2 out of 5 (“slight problems”) in all dimensions. This absolute reduction of 1% (relative reduction of 1.6%) in the proportion of individuals with the best health state was lower than that reported in the population of South Korea and Lombardy (absolute reductions of 4.5, and 5.9%, respectively) [28, 31]. The Canadian and English studies [29, 30] reported greater differences of 9.8 and 8.6%; but as previously remarked, they were not head-to-head comparisons, so this could be explained by other reasons related to the study design, rather than to differences between EQ-5D versions.

This is the first time that redistribution of a large proportion of individuals from extreme to severe problems has been reported in a general population. Depending on the dimension, between 18.3 and 75.7% of individuals reporting extreme problems in the 3 L version moved to level 4 (severe problems) in the 5 L one. The better perceived health in this latter subgroup (VAS mean over 40 in most domains), compared with the subgroup remaining in extreme problems (VAS mean ranging from 29.5 in anxiety/depression to 36.5 in self-care), supports the validity of the redistribution phenomenon observed in the side of the EQ-5D descriptive system indicating poor health. This may indicate that the EQ-5D-5L can measure the health state of individuals with severe (but not extreme) health problems in the Catalan general population better than the EQ-5D-3L. This partly explains why the index mean of the new version was higher (0.89) than that obtained with the traditional version with three levels (0.87). Due to its small sample size (*N* = 600), the South Korean study could not observe this redistribution because there were too few participants on level 3 of EQ-5D-3L (0–6 individuals) [28], while the Italian study did not assess the redistribution [31]. It is important to highlight the low average proportion of inconsistencies between both EQ-5D versions in our study (0.9%), which was comparable to the South Korean general population (1.1%) [28], and lower than that reported among patients with cancer (3.5%) [25] or with chronic conditions (2.9%) [39].

As expected, extending the EQ–5D descriptive system from three to five levels resulted in significantly higher absolute, but slightly lower relative (evenness) discriminatory power. J’ values have also been found slightly lower in some dimensions of EQ-5D-5L in previous comparative studies [37, 39, 40]. The absolute and relative informativity of both EQ-5D versions in our study (0.36–1.37 and 0.21–0.68, respectively) were similar to those reported by Pattanaphesaj et al. [40] (0.12–1.40 and 0.08–0.63), but lower than those observed in others [37, 39]. The relatively good health of people from the Catalan general population could partly explain the lower absolute informativity observed in our study.

The difference observed between EQ-5D-3L and EQ-5D-5L indices for medians and means (SD) merits a comment. The EQ-5D-5L index presented a slightly higher median and mean, but a reduced SD compared with the EQ-5D-3L index. Since the crosswalk 3 L–5 L value set applied to calculate the EQ-5D-5L index had been derived from that originally developed for the 3 L version, these differences may be mainly explained by the increment in the number of levels. For this reason, it is recommendable that national health surveys using the EQ-5D-3L that decide to replace it with the EQ-5D-5L maintain both versions, at least in a random subsample, for a temporary period. Results in these subsamples will allow anchoring results of the two versions, in order to take into account the version effect and correctly monitor the evolution of health along time. Otherwise, changes observed when monitoring populations could be mistakenly attributed to health worsening/improvement instead of measurement differences between versions.

The most prevalent chronic conditions in this sample were low back pain (30%), arthrosis, arthritis or rheumatism (27.8%), and high blood pressure (25.6%), while stroke was the least prevalent with a rate of 2.4% (data not shown). Contrarily to the a priori hypothesis, both EQ-5D versions had an almost identical validity measuring health in individuals who self-reported chronic conditions and with the best health state. This unexpected result is probably explained by the very similar percentage of individuals with the best health state within each specific chronic condition, regardless of the EQ-5D version. Although larger reductions in this percentage were reported in studies of specific conditions such as hepatitis B (21.6 to 16.7%) [26] and surgery patients (30 to 18%) [27], the decline observed in the groups with specific chronic conditions within our sample was ≤3% in all cases. This difference could be due to self-reporting instead of clinical diagnoses.

## Conclusions

The increase of levels provided by the EQ-5D-5L contributed to the validity and discriminatory power of this new version. The group of individuals with poor health was redistributed into different severity levels, while in the EQ-5D-3L they were stuck in the category of extreme problems. The proportion of individuals with the best health state is still very high in the EQ-5D-5L. Nonetheless, results of perceived health VAS support validity of the observed redistribution. Furthermore, consistency between both EQ-5D versions and with results from the 2011 Spanish National Health Survey enhance the reliability of responses from this subset of general population in good health.

Our findings support the validity and discriminatory power of the new EQ-5D-5L for health measurement of the general population. However, it would be advisable to maintain both versions in parallel for a temporary period when introducing the new EQ-5D-5L to a national health survey currently using the EQ-5D-3L version in order to establish an anchor.

## Additional files

Additional file 1: Sensitivity analysis performed with the newly developed Spanish value set obtained through a common composite method of Time Trade Off (TTO) and discrete choice experiments (DCE): Distribution of the EQ-5D-3L and EQ-5D-5L indices (total sample and positive values subsample). (DOCX 15 kb)

Additional file 2: EQ-5D-5L comparison between 5th and 6th waves to assess effect of administering it after EQ-5D-3L. (DOCX 19 kb)

## Abbreviations

CHIS: Catalan Health Interview Survey
IQR: Interquartile range
SD: Standard deviation
TTO: Time Trade Off
