# Supplementary material for: Head-to-head comparison between the EQ-5D-5L and the EQ-5D-3L in general population health surveys
Source: Popul Health Metr. 2018 Aug 16;16:14. doi: 10.1186/s12963-018-0170-8 (PMC6097421; doi:10.1186/s12963-018-0170-8)
Supplement: Supplementary file 1 — Sensitivity analysis performed with the newly developed Spanish value set obtained through a common composite method of Time Trade Off (TTO) and discrete choice experiments (DCE): Distribution of the EQ-5D-3L and EQ-5D-5L indices (total sample and positive values subsample). (DOCX 15 kb) [file 12963_2018_170_MOESM1_ESM.docx]

|  | **EQ-5D-3L**  **(TTO)** | **EQ-5D-5L**  **(TTO&DCE)** |
| --- | --- | --- |
| **Total sample** | N = 7554 | N = 7554 |
| **Theoretical range** | -0.653 , 1 | -0.416 , 1 |
| **Observed range** | -0.653 , 1 | -0.416 , 1 |
| **% with worst health state**  **(95% CI)** | 0.14%  (0.04 , 0.24) | 0.03%  (0 , 0.08) |
| **% with best health**  **(95% CI)** | 61.82%  (59.38 , 64.26) | 60.82%  (58.36 , 63.28) |
| **Mean, SD**  **(95% CI)** | 0.87, SD = 0.25  (0.86 , 0.88) | 0.90, SD = 0.19  (0.89 , 0.91) |
| **Median [IQR]** | 0.93 [0.87 , 0.96] | 0.96 [0.90 , 0.98] |
| **Subsample*** | n = 7305 | n = 7305 |
| **Theoretical range** | 0 , 1 | 0 , 1 |
| **Observed range** | 0.015 , 1 | 0.078 , 1 |
| **Mean (SD)** | 0.90 (0.19) | 0.92 (0.14) |
| **Median [IQR]** | 1.00 [0.88 , 1.00] | 1.00 [0.90 , 1.00] |

**Additional file 1.** Sensitivity analysis performed with the newly developed Spanish value set obtained through a common composite method of Time Trade Off (TTO) and discrete choice experiments (DCE): Distribution of the EQ-5D-3L and EQ-5D-5L indices (total sample and positive values subsample).

***After excluding participants with negative values in any index.**

**Footnote:** The EQ-5D-3L index was calculated with the conventional Time Trade Off preference value set [33].

The EQ-5D-5L index was calculated with the definitive value set [41] obtained using a common composite method of TTO and DCE.
