# Supplementary material for: Head-to-head comparison between the EQ-5D-5L and the EQ-5D-3L in general population health surveys
Source: Popul Health Metr. 2018 Aug 16;16:14. doi: 10.1186/s12963-018-0170-8 (PMC6097421; doi:10.1186/s12963-018-0170-8)
Supplement: Supplementary file 2 — EQ-5D-5L comparison between 5th and 6th waves to assess effect of administering it after EQ-5D-3L. (DOCX 19 kb) [file 12963_2018_170_MOESM2_ESM.docx]

**Additional file 2. EQ-5D-5L comparison between 5^th^ and 6^th^ waves to assess effect of administering it after EQ-5D-3L.**

|  | 5^th^ wave  (3L & 5L together)  July-December 2011 | 6^th^ wave  (5L alone)  January-June 2012 | p-value |
| --- | --- | --- | --- |
| Mobility | N (%) | N (%) |  |
| No problems in walking about | 1645 (84.7%) | 1569 (82.3%) | 0.101 |
| Slight problems in walking about | 102 (5.3%) | 127 (6.7%) |  |
| Moderate problems in walking about | 128 (6.6%) | 118 (6.2%) |  |
| Severe problems in walking about | 57 (2.9%) | 77 (4.0%) |  |
| Unable to walk about | 9 (0.5%) | 16 (0.8%) |  |
| Self-care |  |  |  |
| No problems washing or dressing myself | 1811 (93.3%) | 1773 (93.0%) | 0.778 |
| Slight problems washing or dressing myself | 46 (2.4%) | 52 (2.8%) |  |
| Moderate problems washing or dressing myself | 44 (2.3%) | 31 (1.6%) |  |
| Severe problems washing or dressing myself | 18 (0.9%) | 21 (1.1%) |  |
| Unable to wash or dress myself | 23 (1.2%) | 28 (1.5%) |  |
| Usual activities |  |  |  |
| No problems doing my usual activities | 1711 (88.0%) | 1660 (87.1%) | 0.673 |
| Slight problems doing my usual activities | 69 (3.5%) | 83 (4.4%) |  |
| Moderate problems doing my usual activities | 80 (4.1%) | 83 (4.4%) |  |
| Severe problems doing my usual activities | 30 (1.5%) | 37 (1.9%) |  |
| Unable to do my usual activities | 52 (2.7%) | 43 (2.3%) |  |
| Pain/discomfort |  |  |  |
| No pain or discomfort | 1405 (72.4%) | 1289 (67.6%) | **0.003** |
| Slight pain or discomfort | 201 (10.3%) | 215 (11.3%) |  |
| Moderate pain or discomfort | 224 (11.5%) | 234 (12.3%) |  |
| Severe pain or discomfort | 103 (5.3%) | 148 (7.7%) |  |
| Extreme pain or discomfort | 9 (0.4%) | 21 (1.1%) |  |
| Anxiety/depression |  |  |  |
| Not anxious or depressed | 1615 (83.2%) | 1515 (79.5%) | 0.159 |
| Slightly anxious or depressed | 160 (8.2%) | 163 (8.5%) |  |
| Moderately anxious or depressed | 113 (5.8%) | 146 (7.7%) |  |
| Severely anxious or depressed | 43 (2.2%) | 68 (3.6%) |  |
| Extremely anxious or depressed | 10 (0.5%) | 1. (0.8%) |  |

**In bold: Chi-squared test is statistically significant**
